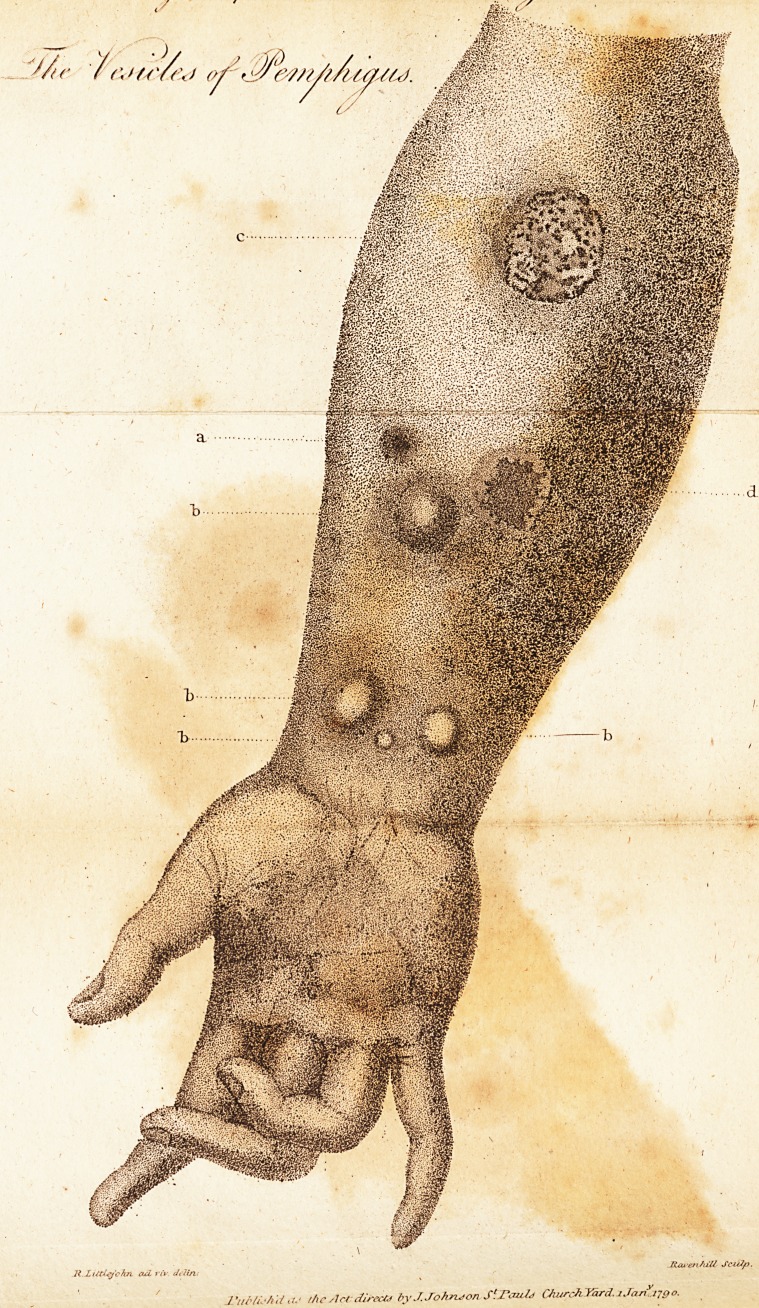# Observations on Pemphigus

**Published:** 1789

**Authors:** Thomas Christie

**Affiliations:** Member of the Medical and Antiquarian Societies of Edinburgh


IV. *Obſervations on Pemphigus. Communicated in a Letter to Dr.* Simmons *by Mr.* Thomas Chriſtie, *Member of the Medical and Antiquarian Societies of Edinburgh.*

*To Dr.* Simmons,

Dear Sir,

THE following eſſay was originally deſigned to be publiſhed as a theſis; but a variety of circumſtances having combined to difappoint my wifhes in profecuting medical ftudies, I believe I fhould not have thought of printing it at all, if the partiality of your friendfhip had not pledged me to the Public by announcing it in the London Medical Journal *. I muft, therefore, requeft that you will accept of it, and give it a place in that ufeful publication, as a fmall teftimony of my efteem for and gratitude to the Editor. The external events of life it is often out of our power to regulate or control; but whatever changes may take place in my future condition, I fhall always retain a partial attachment to medical fcience, and a fincere refpect for its worthy profeffors.

I am, &c.

THOMAS CHRISTIE.

September 1, 1789.

THERE are fome difeafes to which it is difficult to affign a name, either becaufe they are in an incomplete and unformed ftate, or becaufe they are fo complicated with other maladies, that we may fay, with more propriety, that the patient labours under a number

* See Vol. IX. page 310.

of of morbid ſymptoms, than that he is affected with any particular diſeaſe. A wiſh to ſatisfy the curioſity of a patient or his friends, and ſome other reaſons, ſuch as an immoderate attachment to noſology, a want of ſagacity, and a diſtinguiſhing ſpirit, have indeed rendered phyſicians very fond of beſtowing names on diſeaſes, and of comprehending the complaints of their patients under one particular term. Yet it may be made a queſtion, which I ſhall leave to older practitioners to decide, whether, amongſt the complaints that occur in real life, there are not as great a number of this irregular and unfixed kind, as of thoſe to which one can with ſtrict propriety apply the name which diſtinguiſhes any regular and fixed diſeaſe? Some practitioners indeed ſave themſelves from all difficulty, by deſcribing diſeaſes in ſuch a looſe and inaccurate manner, and aſſigning to them ſuch numerous and various ſymptoms, that their terms become quite vague and transferable; ſo that one ſet of ſymptoms, according to their way of going to work, may either denote the firſt ſpecies of one diſeaſe, the laſt of another, or the middle ſpecies of a third. But this is multiplying words without meaning, and loading the memory without informing the judgment.

The importance of the diagnoſtic is, that it influences the practical part of phyſic; for when the phyſician has decided on the diſeaſe, he has recourſe to his accuſtomed mode of cure. It is evident, therefore, that he cannot be too cautious in pronouncing on the firſt, becauſe the nature of things doth not accommodate itſelf to our judgments about them; nor can we avoid committing grievous errors, if we take up words inſtead of facts, and imagine ourſelves extremely wiſe, when in reality we are very ignorant.

Notwithſtanding it is thus difficult to decide on diſeaſes, and to apply with propriety a particular term to a claſs of ſymptoms, yet it muſt be admitted that it is often poſſible; and wherever it is ſo, nothing can be more deſirable.

If the obſcurity that attends inward diſeaſes, and the ſimilarity which ſubſiſts between ſome of their ſymptoms, ſhould render it more difficult and precarious to decide on them, one would, however, imagine, that with reſpect to external complaints, which offer viſible appearances to the eye, it would not be difficult to determine determine the diſeaſe, and refer it to the claſs to which it belongs. This is not, however, the caſe. Cutaneous diſeaſes, though accompanied with eruptions, are as little underſtood among phyſicians as any claſs whatever. This may be, in part, owing to their variety and minuteneſs; but it is probably more owing to the want of attentive and accurate obſervation, and to the confounding various and different things under one name.

A remarkable inſtance of this we have in the word *Scurvy*, which, inſtead of being confined to one diſtemper, is, in the common language of mankind, and even in that of many practitioners of phyſic, applied to denote almoſt the whole variety of diſorders which affect the ſkin: for what eruption may not be ſatisfactorily accounted for by ſaying that it proceeds from a *ſcorbutic humour* *?

To

* "The term *Scurvy*," ſays the judicious Macbride, "is "often indiſcriminately applied, even by medical people, to "almoſt all the different kinds of cutaneous foulneſs; and "this vague way of ſpeaking is owing to ſome writers of the "laſt century, who comprehended ſuch a variety of ſymptoms under this denomination, that there are few chronic "diſeaſes but may, according to this ſcheme, be called a "*ſcurvy*."

To put an end to this confuſion and uncertainty, it is neceſſary that phyſicians ſhould ſtudy the natural hiſtory of diſeaſes with the ſame care that botaniſts ſtudy that of plants, or zoologiſts that of animals. In cutaneous diſeaſes plates ought to be given; for in ſuch caſes the moſt accurate deſcription falls far ſhort of a good drawing.

Of the great work of Linnæus ſome one has obſerved, with a kind of enthuſiaſm, " figuris " non egebat,"—" it needed no plates;" which may be admitted, if meant only as a poetical way of deſcribing its extreme accuracy; but cannot be allowed, if intended to convey a ſober, philoſophical truth. The addition of plates would much aſſiſt the juſtneſs, and ſtill more the quickneſs, of the reader's comprehen-

" *ſcurvy*." — Introduction to the Theory and Practice of Phyſic, 4to. page 615.

In the ſame looſe manner, remarks M. Sauvages, the ancients applied the term *Ophthalmia* to inflammatory complaints of the eyes, from whatever cauſe they originated, or whatever part of that organ they affected: hence they recommended an hundred different remedies of the moſt various and oppoſite kinds for the cure of one diſeaſe: and their advices are of no uſe to us, becauſe we cannot tell in what ſpecies of the diſeaſe the medicine would be of any ſervice.— *Vide* Noſol. Method. Tom. I. p. 86.

ſion of the objects deſcribed: and accordingly Linnæus himſelf, in other parts of his works, and all his ſucceſſors, have employed themſelves in giving views from nature of new plants, as a neceſſary appendix, even to the beſt verbal deſcription of them.

It is for this reaſon that I have judged it proper to accompany the few obſervations I intend to make on a cutaneous diſeaſe, known by the name of pemphigus, with a plate *, copied from nature, in order, if poſſible, to fix the idea of a diſorder, hitherto little underſtood, and to enable future obſervers to collect facts relative to it by promulgating an univerſal knowledge of what is to be deſcribed †.

* In the plate, which is annexed to this paper, *a.* refers to a veſicle beginning to make its appearance; *b. b. b. b.* are veſicles of various ſizes come to maturity; *c.* is a veſicle burſt, and incruſted over; and *d.* refers to the appearance on the ſkin which laſts for ſome time after the cruſt is gone.

† Since I wrote theſe remarks I have had the pleaſure to find that Dr. Willan, an ingenious phyſician in London, has taken up the ſame idea reſpecting the neceſſity of having drawings made of the appearance of cutaneous diſeaſes which I have ſo ſtrongly recommended in this paper. He has indeed proceeded a great way in executing this plan, and I hope will ſoon favour the Public with a ſplendid work on the ſubject.

A minute

A minute detail of the obſcure opinions of remote ages, relative to the diſeaſe before us, would rather ſerve to diſplay learning than to produce any uſeful conſequence. I ſhall, therefore, begin with M. Sauvages, the publication of whoſe excellent noſology forms an æra in medical hiſtory; and I ſhall only notice earlier writers as they have been referred to or quoted by him.

Sauvages places pemphigus amongſt the *exanthematicæ*, or eruptive diſeaſes, which conſtitute the firſt order of his third claſs*, and thus defines it.

"PEMPHIGUS, *pemphigos* & *pemphigodes pure-*

"*tos* Galen. 6a epidemiar. *hydroa* Car. Piſonis;

"ſic dicta a πέμφιξ, bulla vel phlyctæna;

"bulloſa febris, nouvell. claſs. fievre veſicu-

"laire †."

In the laſt edition of Dr. Cullen's Synopſis Noſologiæ Methodicæ it is placed in the firſt claſs (pyrexiæ); order iii. (exanthemata); gen.

* Morbi Inflammatorii ſeu Phlegmaſiæ.

† Vide Noſol. Method. 4to. Amſtel. 1768. Tom. I. p. 430.

34, and is defined " typhus contagiosa," a contagious low fever *.

Dr. David Stewart, physician in Aberdeen, met with one case of it in the hospital there, and published an account of it in Dr. Duncan's Medical Commentaries for the year 1778, page 79.

Burserius, a learned and judicious physician at Milan, in the second volume of his Institutiones Medicinæ practicæ, printed in 1785, treats of pemphigus amongst the febrile exanthemata.

Since that time Dr. Stephen Dickson, professor of physic at Dublin, has published a very interesting paper on this subject in the first volume of the Irish Transactions, page 47.

From these five sources I shall collect what is known relative to the history of this disease, comparing the different accounts together, interspersing my own observations, and adding an account of a case that occurred at the Westminster General Dispensary in May, 1788.

In this disease, says Sauvages, " the inflam-

" mation is for the most part acute, and is

* Vide Synops. Nos. Meth. Ed. 4. 8vo. Edin. 1785. Tom. II. p. 148.

" attended with an eruption of large pellucid " bliſters or veſicles, filled with a yellowiſh " ſerum, and ſcattered over the ſkin. It dif- " fers greatly from the miliary fever, in which " there are indeed veſicles, but not larger than " a grain of millet; and alſo from the variola " cryſtallina (chicken pox), where the veſicles " are filled with pus, or formed by the junc- " tion of ſeveral ſmall puſtules into one large " one. In pemphigus the veſicles are about " the ſize of an hazel nut, ſometimes larger, " rarely leſs, and filled with a ſerum of a light " yellow colour.

" I am pretty certain," he adds, " that this " is a new diſeaſe, as no diſtinct account of it " occurs in the Greek or Arabian writers; how- " ever, it is not ſo rare, but that I have met " with ſix caſes of it."

Of the opinion that pemphigus is a new diſeaſe I ſhould be inclined to doubt. The diſeaſe may not be new, although we have no accounts of it preſerved in the ancients. There were few among them who were accurate obſervers; little had then been done in diſtinguiſhing ſimilar diſeaſes from each other; many of their works have not come down to us, and in thoſe we have there are ſeveral diſeaſes whoſe name alone is known to us, but of whofe nature we are either uncertain or altogether ignorant *.

Hippocrates, in the fixth book of his epidemics, mentions Πυρετοὶ πεμφυγώδεες, tranflated, in the edition of Charterius, *Febres acutæ*; and Galen, in his comment on this paffage, gives different fenfes to the word, and takes notice of πυρετοι μετὰ φλυκταινων, febres cum tuberculis.—Hippoc. et Galeni, Op. ed. Charterii, fol. Tom. IX. p. 382 †.

But it is not fufficiently clear what difeafe was meant by thefe ancient writers, and therefore nothing can be built on this ‡.

The

* This fact was well known to M. Sauvages himfelf, and he adduces it as a proof of the neceffity of an accurate defcription of difeafes in his addrefs *Lectori Philiatro*: —"Fruftra "nominantur morbi, nifi defcriptione fixa determinentur; "defcriptionis defectu ignoramus hactenus quid fint morbi "plurimi ab Hippocrate nuncupati, ut *typhus*, *pachy*, *avante*, "*phrontis*, *phoenicie*, *leuce*, *hippouris*, *pherea*; quid Plinii *ge-* "*murfa*, aliique innumeri:" and, he adds, as another inftance of the vague manner in which the ancients fpoke of difeafes, that we cannot tell with certainty what is meant by the morbus Scitharum of Hippocrates, by the magni fplenes, morbus niger, and morbus ructuofus.—Nofol. Method. I. 86.

† See alfo the Oecon. Hippocr. by Foefius, p. 297.

‡ "Verum quid hoc nomine intellexerit Hippocrates, nec "Galenus in commentario certo definire aufus eft, nec alii

 "interpretes

The firſt ſpecies of it Sauvages calls *pemphigus major* of Chriſt. Seligerus, the hydatides of C. Piſo (Obſ. 147, 149), the veſicular catarrhal fever of Delius (amoenitates medicæ), and ſays it is accompanied with conſtant acute fever, on the ſecond or third day of which the veſicles ariſe; that, when they break, they are ſucceeded by large dark red ſpots, with black edges; and that the diſeaſe laſts about a fortnight. He had met with ſome caſes of it in the hoſpital, and in two there was no fever. One of the caſes that occurred to him was fatal.

On this Dr. Cullen obſerves, that every degree of credit is due to what M. Sauvages mentions, from his own experience, or from Piſo; but nothing to the authority of Seligerus, "tenuis certe judicii hominis."—(Synopſ. Noſ. Meth. T. II. p. 149.)

Piſo, ſays Dr. Dickſon, (Iriſh Tranſactions, page 49) accurately deſcribes the genuine pemphigus in the caſe of Egmont de Rinach. He terms it hydatids, and ſays it occurred to him

"interpretes ſatis explicarunt. Hinc jure adhuc inter eos "diſceptatur."—Vide Burſerii Inſtit. Med. Pract. Tom. II. p. 115.—He refers for farther information to Caſpar a Rejes Elyſ. jucund. quæſt. camp. Quæſt. 68. n. 7.

frequently; frequently; but the Doctor suspects he confounded it with the chicken pox, and with several erythematous affections; adding justly, that though an industrious observer and candid man, he was not an acute nosologist.

To this first species Dr. Cullen refers the *exanthemata serosa* of Piso, Obs. 150, and the *febris pemphygodes*, Ephem. Germ. D. I. A. VIII. Obs. 56.

The second species mentioned by M. Sauvages is the *pemphigus castrensis*, or camp pemphigus, described by M. Thierry, a French physician, who says it raged at Prague in 1736, and baffled all the art of physic, till some one (whom M. Thierry stiles a great practitioner) suspecting that the vesicles were analogous to those produced by a blister, and took their origin from an acrid ferment, (for such was the language of the times) prescribed bezoartic vinegar to the sick, and recovered them all, while the other physicians lost almost every patient *.

On

* "Il regnoit en 1736, une maladie fort contagieuse à

" Prague; les resources et les reflexions de la faculté etoient

" epuisées. Toutes les methodes echouoient contre la fero-

" cité du mal. Un grand praticien de cette ville, qui n'etoit

" pas

On this relation Dr. Cullen juſtly thinks that no dependence can be placed. So violent a diſeaſe cured infallibly by ſo impotent a remedy, "meam fidem omnino ſuperat," ſays he. Indeed if we acquit the veracity of M. Thierry, we cannot at leaſt pay any compliment to his judgment.

To this ſpecies Dr. Cullen refers the *febris ſyneches, cum veſiculis per pectus et collum ſparſis,* of Dr. Morton, App. ad exerc. ii. Burſerius, however, doubts whether Morton's fever, which he has only mentioned, but not deſcribed, can be referred to this diſeaſe.

The third ſpecies noticed by M. Sauvages is the *pemphigus Helveticus*, or Swiſs pemphigus of Dr. Langhans, (Acta Helvetica, Vol. II. page 260, and in Beſchreibung des Siementhals, Zu-

" pas plus avancé que les autres, conſiderant un jour les veſi-

" cules qui s'elevoient ſur la peau, (j'en ai vû d'auſſi groſſes

" que des noiſettes) il trouva qu'elles reſſembloient a celles

" que forment les veſicatoires ; il ſoupçonna que le ferment

" acre qui dominoit dans les humeurs, pouvoit être du même

" caractere que celui que fourniſſent les mouches cantharides;

" d'après cette idee, il ordonna le vinaigre bezoardique à ſes

" malades, et les ſauva tous, tandis qu'il n'en echappoit preſ-

" que aucun entre les mains des autres medecins."—Medecine Experimentale, ou reſultat de nouvelles Obſervations pratiques et anatomiques. 8vo. Paris, 1755, p. 134.

rich, rich, 1753) an epidemic diſeaſe, which began in Switzerland towards the end of winter 1751, and raged violently: it was attended with nauſea and a cold, but no hot fit. The puſtules chiefly affected the throat, both internally and externally, though they appeared alſo on other parts of the body. They were of the ſize of a nut, and, when opened, diſcharged a fœtid yellowiſh ichor. This diſeaſe was often fatal.

Dr. Cullen calls it an ambiguous diſorder, and thinks that perhaps it was nothing more than a malignant ſore throat: however, I am inclined to regard it as a ſpecies of the true pemphigus.

To this ſpecies Dr. Cullen refers the veſicatory fever of Macbride, (Meth. Introd. to the Theory and Practice of Phyſic, page 389) whoſe account contains nothing more than has been already communicated to the reader.

The fourth ſpecies enumerated by M. Sauvages, and to which he refers the *bulloſa febris cum dyſenteria* of Morton, (Pyretolog. Append. p. 163) is the *pemphigus Indicus*, or Indian pemphigus, a name he gives to a diſeaſe mentioned by Bontius in his Treatiſe de Medicina Indorum; but upon looking into that work I find in it nothing more than a brief account of a

ſingle

ſingle and fatal inſtance of what the author calls an ardent fever, attended with dyſentery and an eruption of puſtules and veſicles on different parts of the ſurface of the body. Theſe veſicles are ſaid to have been filled with a greeniſh pus, and to have terminated in malignant ulcers; but the deſcription* given of them is too imperfect to enable us to form any accurate idea of the real nature of the diſeaſe.

The fifth and laſt ſpecies mentioned by M. Sauvages is the *pemphigus Braſilienſis*, the pemphigus of Brazil, ſaid to be occaſioned by touching a ſerpent found in that country; and for an account of which he refers his readers to Father Bougeant's Obſervations curieuſes ſur la Phyſique, Tom. I., publiſhed in 1730. Of

* "*De febre ardenti, dyſenteriâ, ulceribus malignis,* &c. "*Obſ.* — Reverendus ac doctiſſimus vir Joannes Cavallerius, "verbi divini præco, correptus eſt febre ardente, ſuperveniente dyſenteria atrabiliaria, quæ cum per aliquot dies "continuaſſet, eruperunt ſub axillis, in tergo circa lumbos, "et in inguinibus, etiam in collo, puſtulæ ac veſicæ quædam, "plenæ ac diſtentæ pure viridi, et ſubjectam cutem ad carnem "uſque erodente, quæ nobis, prima facie, ſpem criſeos faciebant: ſed dyſenteria non ceſſante ac febre, cum phrenitide ingraveſcente, probiſſimus ſimul, ac doctiſſimus juvenis, ex hac vita ad cœlos raptus eſt."—*Vide* Bontii Obſ. de Medicina Indorum. 4to. Pariſiis, 1645. p. 38.

this this fpecies, however, nothing certain can be concluded.

Dr. Cullen's definition of pemphigus is as follows*:

"Typhus contagiofa. Primo, fecundo, aut "tertio die, in variis partibus veficulæ, avel-"lanæ magnitudine, per plures dies manentes, "tandem ichorem tenuem effundentes. — A "contagious low fever. On the firft, fecond, "or third day, veficles of the fize of a hazel "nut appear on various parts of the body: "they remain for many days, and at laft pour "forth a thin ichor."

The Doctor owns, that as he had never met with a cafe of the kind himfelf, and found little about it in the writings of phyficians, he had been obliged to take almoft his whole account from Sauvages. He makes fome remarks, already noticed, on the authorities cited by that author; feems to think pemphigus, in all cafes, a fymptomatic difeafe; and adds, in a note, that, fince he wrote them, Dr. Home had brought a man to him who was flightly feverifh, and had had an eruption, firft on his arms, and then over all his body, of puftules of

* Vide Synopf. Nofol. Method. Tom. II. p. 148.

the ſize of a nut, filled with a watery humour, which in two or three days broke and healed up. He adds, that this fever aſſumed no particular type, and ſoon went off, not being at all contagious.

The ſynonyma, according to Dr. Cullen, are to be found in the *pemphigus* of Sagar, the *morta* of Linnæus, and the *febris bulloſa* of Vogel. From their accounts, however, nothing can be added to what we have already given.

We come next to the caſe related by Dr. Stewart, the ſubject of which was a ſoldier of the ſeventy-third regiment, who was received into the hoſpital at Aberdeen. He had been afflicted three weeks before with the meaſles, which, on the ſecond day of the eruption, had ſuddenly diſappeared in conſequence of his having been expoſed to cold; and ten days after he perceived, on the inſide of his thighs, an eruption of ſmall red ſpots, which, increaſing in ſize, became filled with ſerum, and gradually ſpread over all the ſkin. Some of theſe veſicles diſcharged pus, and others a bloody ichor. The patient was beſides affected with ſickneſs, laſſitude, oppreſſion about the præcordia, thirſt, ſore throat, with difficulty of ſwallowing; his tongue was foul; his ſkin hot and feveriſh; his his pulſe beat from 110 to 120 ſtrokes in a minute; he was coſtive; and his eyes appeared languid.

Dr. Dickſon, who has taken notice of this caſe in his obſervations on pemphigus, thinks we may infer from it that the nature of the fluid contained in the veſicles, though at firſt a pure ſerum, may be ſo altered in the courſe of the diſeaſe, by its own fermentation, or admixture with other fluids, from their veſſels being broken down, as to ceaſe from being a diagnoſtic of the diſeaſe.

Burſerius devotes the ſecond volume of his Inſtitutiones Medicinæ practicæ to febrile exanthemata; and in the ſeventh chapter of that volume we find him treating *de pemphige recentiorum ſive morbo phlyctænoide.*

I had finiſhed my obſervations on this diſeaſe in MS. before I had an opportunity of conſulting his work. I was, therefore, much pleaſed to find that he agreed with me in dividing the diſeaſe into two kinds only. The firſt, he ſays, is mild, either without fever, or with a very ſlight degree of it, the puſtules riſing from the firſt to the fourth day, and going off before the ſeventh; while the laſt is malignant, often epidemical and contagious.

He does not venture to affirm that the diſeaſe is always mild, even when without fever; and relates the caſe of an old man who was ſuddenly ſeized with an eruption of large watery puſtules, and, without fever, died in a few days.

The pemphigus Helveticus, he thinks, was either another diſeaſe, or at leaſt complicated with angina. The laſt is moſt probable. He contends that pemphigus is not by any means always ſymptomatic, and that no caſe of it is without danger, as the tendency to putrefaction is conſtantly very conſiderable.

Dr. Dickſon begins his "Obſervations on "Pemphigus*" with remarking, that it is a diſeaſe of rare occurrence. This may be partly true; but I apprehend it has not hitherto been ſufficiently known to be obſerved when it did occur. He propoſes to alter Dr. Cullen's definition in the following manner: — "A fever, "accompanied with the ſucceſſive eruption "from different parts of the body, internal "as well as external, of veſicles about the ſize "of an almond, which become turgid with

* See the Tranſactions of the Royal Iriſh Academy, Vol. I. 4to. Dublin, 1787; and the ninth volume of this work, page 309.

"a faintly

" a faintly yellowiſh ſerum, and in three or " four days ſubſide."—From this definition it is evident he differs from Dr. Cullen in five points.

1. He is by no means convinced that the diſorder is contagious.

2. He finds that new veſicles ariſe, not only on the firſt, ſecond, or third, but on every day of the diſeaſe.

3. He has never known them to remain many days.

4. The fluid they contain does not appear in general to be an ichor or ſanies, but a bland, inodorous, inſipid ſerum. And

5. Inſtead of being poured out, it is moſt commonly abſorbed into the ſyſtem.

Six caſes, it ſeems, have occurred to Dr. Dickſon, two of which he has particularly deſcribed. The firſt was that of a woman under the care of Dr. Gregory in the Infirmary at Edinburgh in 1783. In this patient the menſes had been obſtructed for two years and a half, " during which period ſhe had been thrice " before attacked with the ſame diſorder, which " had each time ſupervened upon a vomiting " of blood. Her ſkin was generally cool, and

" her

" her pulse, though weak, never much increased

" in frequency."

The next is that of a married lady in Ireland, aged twenty-three, who, after being ill during fourteen days of a low fever, was seized with pains in her back, head-ach, and sickness. The next day her pulse became frequent, and she complained of a sore throat. On the third day she complained of a smarting, tingling kind of pain in her tongue and inside of her mouth. She was thirsty and costive, and had her taste vitiated. On the fourth day a pellucid vesicle of about an inch long, and half an inch broad, turgid with a faintly yellowish fluid, appeared; a smaller one appeared on the inside of the left cheek. On the fifth, sixth, and seventh days the symptoms continued; new vesicles appeared externally on the cheek and neck; and she had no sense of taste. On the eighth the vesicles on the tongue and mouth disappeared. The whole inside of the throat continued painful, and deglutition difficult. On the ninth the cuticle on the parts formerly occupied by vesicles within the mouth, cracked and peeled off, leaving the parts beneath raw and sore. Deglutition had now become so painful, that she refused medicine, food, and even even drink. On the tenth new veſicles appeared on the abdomen. On the thirteenth ſhe vomited ſome blood along with a doſe of the bark; ſeveral veſicles of the ſize of a pea aroſe on the hypogaſtric region of the abdomen, one on the labia pudendi, and two on the left thigh. On the fourteenth ſhe had two looſe ſtools, much intermixed with blood, and complained of great ſoreneſs of her belly, increaſed by preſſure. After the fifteenth ſhe recovered daily.

Dr. Dickſon obſerves, that no former author has mentioned the circumſtance of the veſicles taking poſſeſſion of the *internal* parts of the body, and proceeding in ſucceſſion, ſome riſing whilſt others decayed, through the whole ſurface of the alimentary canal.

If, however, we admit the diſeaſe deſcribed by Dr. Langhans to be a ſpecies of pemphigus, which I think we muſt do, as it was attended with febrile ſymptoms, and the puſtules appeared on every part of the body, and were of a large ſize, it will follow that this circumſtance was obſerved firſt by him; for he ſays that the veſicles attacked the fauces, pharynx, and, as it would ſeem, other parts alſo; and that the event of the diſeaſe depended upon

4 the the patient's conſtitution having power enough to throw out all the matter from the internal to the external parts of the body. Indeed I apprehend that the appearance of the veſicles on internal parts denotes only a more virulent ſtate of the diſeaſe, ſuch as was that in Switzerland deſcribed by Dr. Langhans, and that in the two caſes mentioned by Dr. Dickſon. In its mild form the diſorder attacks only the ſmall veſſels of the external ſkin, which, owing to ſome peculiarity of their ſtructure, or rather to their diſtance from the ſeat of life, are more expoſed to its influence. This is analogous to what we obſerve in other caſes. Mr. Hunter obſerves, that the ſkin and cellular membrane are extremely ſuſceptible of the ſuppurative ſtage of inflammation, but that internal and deep-ſeated parts reſiſt it long: hence extraneous matters taken into the ſtomach, though they irritate and inflame, ſeldom occaſion ſuppuration; and when, by their acuteneſs or gravity, they pierce through, as in the caſe of pins and bullets, the ſuppuration does not take place till they come near the ſurface of the body. The wiſdom of this law of nature is evident; for if every irritating cauſe could produce ſuppuration in the internal parts as eaſily as it does at the ſurface, a moſt numerous and fatal train of evils would be the conſequence.

I come now to deſcribe a caſe which I myſelf had an opportunity of obſerving at the Weſtminſter General Diſpenſary, and which, though of a milder nature, was a decided caſe of pemphigus.

The patient, Hannah Scott, aged thirty years, and ſervant to Mr. David Jones, of Little Vine Street, Piccadilly, was admitted, under the care of Dr. Simmons, on the 17th of May, 1788. She had, for three months, been occaſionally ſubject to ſickneſs at the ſtomach and head-ach, attended with a ſenſe of weakneſs and laſſitude. About a fortnight before ſhe was admitted at the Diſpenſary the ſickneſs had increaſed, ſhe had become feveriſh, and ſome puſtules had begun to appear on the fore part of her left arm. At firſt they had very nearly the appearance of the ſmall pox. By degrees they became larger, and were filled with a watery yellowiſh liquid. The exertions ſhe was obliged to make at her work uſed to burſt them; but after diſcharging their contents they very often filled again in the courſe of a night; and this proceſs was repeated ſeveral times. New ones alſo appeared; and on the day we firſt ſaw her at the Diſpenſary ſhe had one veſicle, as large as a nut, on her right ſhoulder, one at the pit of the ſtomach, one near the point of the little finger, and about twelve on the arm: they were very ſore, and the ſkin around them was a good deal inflamed. She thought her complaints a little relieved ſince the eruption: however, ſhe was ſtill weak and feveriſh, her tongue was whitiſh, and her pulſe 120. Dr. Simmons, who pointed out the diſeaſe to me as a clear and ſtriking inſtance of pemphigus, preſcribed three grains of calomel to be taken at night, and an ounce of Glauber's ſalt in the morning.

May 22. Her occupations in the family had prevented her from calling at the Diſpenſary; there was, as yet, no alteration in her complaint; and as the menſes (which had returned pretty regularly during the whole of her illneſs) began this day, ſhe was unwilling to take any more medicines during their continuance.

May 24. We ſaw her again, but no change worth noticing had taken place. The menſes ſtill continued.

May 26. Freſh puſtules were to be ſeen in different parts, eſpecially on the leg. Some of the former ones, when they broke, had diſ-

charged a yellowifh fluid tinged with blood. As fhe complained of ficknefs and head-ach, and her pulfe was ftill at 120, fhe was directed to take two table-fpoonfuls of a mixture compofed of two grains of emetic tartar and an ounce of Glauber's falt diffolved in eight ounces of water, and to repeat this dofe, at proper intervals, till it fhould operate by ftool.

May 28. Several ftools had been procured by the medicine laft prefcribed. The ficknefs and head-ach had fubfided; but the pulfe was ftill at 110. She was directed to repeat the antimonial purgative on the 29th.

June 2. The puftules on the arm, after breaking, had moftly healed up, after being covered with a cruft or fcale. The new cutis appeared darkly reddifh and gliftening. Two new puftules appeared on the ancle. She was ordered to repeat the calomel and Glauber's falt.

June 6. A puftule appeared on the lip; but, after the ftricteft inquiry, I could not find fhe had had any on the tongue, infide of the mouth, or any internal part.

June 9. She was evidently a good deal better. The pulfe was now reduced to 100, and the veficles were going off. She was directed to repeat the calomel and Glauber's falt.

June 11. She continued better. The medicines were repeated.

June 27. Nothing particular had occurred till this day, when ſhe had an eruption of ſmall pimples, which might perhaps be conſidered as a proof of her being cured, as they ſhewed that the ſpecific action of the veſſels of the ſkin was changed. Her pulſe was now reduced to 88, and ſhe was free from complaint; but the calomel and Glauber's ſalt were, at her own requeſt, again repeated. After this ſhe took no more medicine, and on the 4th of Auguſt, when ſhe came to the Diſpenſary to return thanks, ſhe was in perfect health.

As this patient ſlept alone, and was the only maid ſervant in the family, we could not judge from her caſe whether the diſeaſe be contagious or not. M. Sauvages ſays nothing with reſpect to this. Dr. Cullen terms it a contagious low fever. Dr. Stewart obſerves, that no perſon laboured under the ſame complaint as his patient, either in the part of the country from which he came, or during his reſidence in Aberdeen. Dr. Dickſon is of opinion that it is not infectious, becauſe all the well-atteſted caſes of it are ſolitary inſtances; and, to confirm his opinion, I ſhall mention, that, ſo far as I could learn, learn, no perſon in the neighbourhood was affected with pemphigus at the ſame time as Hannah Scott, nor did ſhe communicate it to any one.

It appears, however, that the pemphigus Helveticus of Dr. Langhans was extremely infectious; for he ſays that as ſoon as one of a family took it, all the reſt became affected in a ſhort time. This circumſtance may lead us to a new diviſion of the ſpecies of the diſeaſe, and, inſtead of the *pemphigus major*, *caſtrenſis*, and *Helveticus* of M. Sauvages, we may conſider the diſeaſe as exiſting only under two forms, that of *pemphigus ſimplex* and *complicatus*, where it is combined with ſore throat, or ſome other malignant diſorder. Both of them, and eſpecially the laſt, ſeem to vary much with reſpect to mildneſs and malignity.

The diviſion I have made of pemphigus, into two kinds, correſponds exactly with Dr. Cullen's definition of another cutaneous diſeaſe, the ſcarlatina, which he has deſcribed as exiſting in two forms; one ſimple, when there is only an efflorefcence on the ſkin; and another, which he calls *cynanchica*, and Dr. Withering *anginoſa*, when it is attended with an inflammation of the throat.

I truſt it will not be alledged that I have ſimplified the matter too much by reducing the diſeaſe to two kinds. The facts, when properly conſidered, ſeem to me to lead fairly to ſuch a concluſion. In other reſpects, I own, I am ſomewhat inclined to prune the branches of noſology; firſt, becauſe minute diſtinctions in the hiſtory of diſeaſes tend to little practical uſe, the treatment being generally nearly the ſame; and, ſecondly, becauſe the varieties obſervable in them are often more the effect of different conſtitutions than of a different diſeaſe.

Before I conclude the hiſtory of the diſeaſe, I ſhall remark, that there are other eruptive diſorders which may be miſtaken for pemphigus. Sauvages ſays we may diſtinguiſh it from the miliary fever and chicken pox by the larger ſize of the puſtules, and their containing only ſerum. It appears, however, that in the courſe of the diſeaſe they ſometimes contain pus. Dr. Cullen mentions, that, after he had written the obſervations, we have juſt noticed, on this diſeaſe, his colleague, Dr. Home, ſent a man to him who had a ſlight fever, and an eruption of large veſicles, firſt on his arm, and then on other parts of his body. The fever ſhewed no peculiar character, was not contagious, and after two or three days the veficles burft, and, having poured forth a watery fluid, healed up. Very probably this was a flight cafe of what I have called pemphigus fimplex.

A cafe occurred to Dr. Dickfon, when at Edinburgh, of a woman who had fome veficles on the eyelid and eyebrow, which led Dr. Gregory to fuppofe that it was a beginning pemphigus; but as fhe never was affected with any degree of fever, Dr. Dickfon confidered it as merely a local complaint. It was cured in a few days.

Soon after Hannah Scott came to the Difpenfary we had another patient, a young woman aged feventeen, whofe cafe I thought at firft might perhaps be pemphigus. After a fortnight's illnefs fhe was affected with head-ach and ficknefs, which were followed by an eruption of puftules, filled with pus, on her arms, but chiefly on her fhoulder and left breaft; however, I became convinced that I was wrong; for although one or two of the puftules refembled fomewhat, in fize and appearance, thofe of pemphigus, the moft of them were very fmall, became confluent, and after after burſting never filled again: beſides, after the eruption, her general health was very good.

*Of the Treatment of the Diſeaſe.*

"The general ſymptoms of weakneſs and

" tendency to putrefaction," ſays Dr. Dickſon,

" obviouſly point out the proper treatment.

" When the veſicles ſeize on the inward parts,

" irritation muſt be guarded againſt by opiates,

" demulcents, and gentle laxatives; nouriſh-

" ment muſt be ſupplied, and the grand reme-

" dies, bark and wine, eſpecially the laſt, muſt

" be ſeduloully adminiſtered."

Burſerius recommends acids and antiſeptics, particularly the Peruvian bark. He would open the puſtules, leſt the acrid humour ſhould penetrate farther: but he agrees with Vogel in prohibiting medicines which repreſs or dry up the humour. When complicated with ſore throat, he directs the remedies uſed in that diſorder to be applied to.

Dr. Stewart ſnipped the largeſt of the veſicles, and dreſſed them with ung. e lap. calamin., and preſcribed an emetic conſiſting of a ſolution of antimonial tartar, which alſo procured the patient two looſe ſtools. For drink he recommended water gruel acidulated with lemon lemon juice. Next day he ordered half an ounce of a mixture, of two parts of decoction of Peruvian bark and one part port wine, to be taken every three hours. As this seemed to agree with the patient's stomach, the bark in powder was afterwards tried, and half a drachm given every three hours in an ounce of port and water. By this treatment, in about fourteen days, he got quite well.

Dr. Dickson, in his first case, obtained a cure by a liberal use of bark and wine. The second was a much more difficult and dangerous case. The Doctor began with an emetic, and directed the patient to bathe her feet in warm water. Finding her costive, he ordered a clyster, and then gentle laxatives. When her throat became sore, he used tincture of roses as a gargle. Soon after he tried the bark, changed the tincture of roses for an emollient gargle, and recommended imperial for common drink. The patient, however, after being a little relieved, grew worse again, her breath became fœtid, she lost the sense of taste, and even had some degree of delirium. He then ordered an ounce of decoction of bark and half a drachm of salt of tartar to be taken every two hours, and immediately after it half an ounce of the

 same

ſame decoction mixed with ſix drachms of lemon juice; with cyder and porter for common drink. When it appeared that the veſicles had taken poſſeſſion of the lower parts of the alimentary canal, by her diſcharging a little blood both upwards and downwards, he had recourſe to caſtor oil and anodyne clyſters. In three weeks from the time Dr. Dickſon began to attend her ſhe got well of the diſeaſe, and had no complaint but weakneſs, which a little country air completely removed.

In the caſe of Hannah Scott, at the Weſtminſter General Diſpenſary, we purſued, as the reader hath ſeen, a different plan, and the good effects of it were very obvious. The antimonial cathartic, by evacuating the bowels and favouring the determination to the ſkin, ſoon removed the ſickneſs and head-ach; and the patient from the firſt felt herſelf relieved by the operation of the calomel and Glauber's ſalt.

The other ſpecies of the diſeaſe, which I have called pemphigus complicatus, is no where mentioned but in the deſcription of Dr. Langhans already noticed. After bleeding the patient freely once or twice, they applied a large bliſter to the forehead, and ſurrounded the neck with a poultice of bread and milk, which was removed removed every two hours. At the ſame time they preſcribed diaphoretics to be taken internally, and when the patient began to recover he was purged with Epſom ſalt.

## Figures and Tables

**Figure f1:**